# Evolution of Portable Sensors for In-Vivo Dose and Time-Activity Curve Monitoring as Tools for Personalized Dosimetry in Molecular Radiotherapy

**DOI:** 10.3390/s23052599

**Published:** 2023-02-26

**Authors:** Lidia Strigari, Raffaella Marconi, Elena Solfaroli-Camillocci

**Affiliations:** 1 Department of Medical Physics, IRCCS Azienda Ospedaliero-Universitaria di Bologna, Via Giuseppe Massarenti 9, 40138 Bologna, Italy; 2 Scientific Direction, IRCCS Regina Elena National Cancer Institute, Via Elio Chianesi 53, 00144 Rome, Italy; 3 Medical Physics Unit, Bambino Gesù Children’s Hospital, IRCCS, P.zza Sant’Onofrio 4, 00165 Rome, Italy

**Keywords:** time-activity curve, in-vivo dose monitoring, individual absorbed dose, nuclear medicine treatment personalization

## Abstract

Treatment personalization in Molecular Radiotherapy (MRT) relies on pre- and post-treatment SPECT/ PET-based images and measurements to obtain a patient-specific absorbed dose-rate distribution map and its evolution over time. Unfortunately, the number of time points that are available per patient to investigate individual pharmacokinetics is often reduced by limited patient compliance or SPECT or PET/CT scanner availability for dosimetry in busy departments. The adoption of portable sensors for in-vivo dose monitoring during the entire treatment could improve the assessment of individual biokinetics in MRT and, thus, the treatment personalization. The evolution of portable devices, non-SPECT/PET-based options, already used for monitoring radionuclide activity transit and accumulation during therapy with radionuclides (i.e., MRT or brachytherapy), is presented to identify valuable ones, which combined with conventional nuclear medicine imaging systems could be effective in MRT. External probes, integration dosimeters and active detecting systems were included in the study. The devices and their technology, the range of applications, the features and limitations are discussed. Our overview of the available technologies encourages research and development of portable devices and dedicated algorithms for MRT patient-specific biokinetics study. This would represent a crucial advancement towards personalized treatment in MRT.

## 1. Introduction

New radioactive agents and radiopharmaceuticals have been introduced in recent years to treat benign or malignant lesions, both inflammatory diseases. The current state of the art entails the administration of empirically fixed levels of radiation, in some cases modified according to patient weight or body surface area, irrespective of the radioactive distribution or patient status.

Nevertheless, an effort is being made to introduce informatic tools in daily routines which can provide dosimetric follow-up of the patients using routine images [1]. Moreover, several commercial treatment planning systems are now available for radionuclides (RNT) or for Molecular Radiotherapy (MRT) [2].

At the same time, dose-effect relationships have been reported for treatments with RNT or MRT, indicating the possibility of personalizing activity administration based on patient-specific radiotracer kinetics and calculated dose distribution [3]. For this reason, the adoption of fixed activities can lead to under and over-treatments.

Of note, accurate Internal Radiation Dosimetry (IRD) of MRT is not an easy task as the dose delivery depends on several interconnected key factors, such as radionuclide physical properties, radio-compound biokinetics and individual anatomical peculiarities. In addition, the amount and rates of radiopharmaceutical uptake and excretion can greatly fluctuate from patient to patient [4].

According to specific guidance [5,6], patient-specific dose distribution at the organ/target level and voxel level can be determined based on SPECT or PET images and the addition of multiple planar images collected at several time points. Collection and analysis of these images enable the estimation of activity distribution in different target/tissues/voxels at different time points (e.g., 2, 24, 48, 72 h from injection) and the evaluation of its evolution over time. This approach requires the time-integrated activity (TIA) assessment to calculate the absorbed dose-rate and dose map. Due to broad individual uptake and clearance, an accurate sampling of dose rate maps for each patient is desirable in order to determine the absorbed dose precisely.

Several approaches have been proposed to identify the optimal sampling schedules for dosimetry and improve accuracy and precision in TIA determination for radionuclide therapy [7]. However, several reasons limit the time points in which SPECT or PET images can be collected, including patient compliance and the availability of systems to collect images for dosimetric purposes, especially in busy clinical departments [8]. In addition, the accuracy of TAC determination is decreased by patient repositioning on the scanner bed, which increases uncertainty in identifying the volumes of interest (VOIs). Furthermore, SPECT imaging quantification depends on the accuracy of relative calibration that might be affected by filling and voiding of the bladder in MRT patients, which after the administration of therapeutic dosage might be affected by detector saturation phenomena. Indeed, the image acquisition timing and the method used to interpolate data can influence pharmacokinetic reconstruction and organ clearance trends, leading to differences greater than 25% [9,10].

For the reasons herein represented, treatment personalization demands new strategies to quantitatively determine or estimate the absorbed dose of target and normal tissues in order to define the activity to be injected. In other words, we highlight the importance of assessing the activity biodistribution (including the uptake and its evolution over time) as input for an accurate and personalized dosimetry.

Alternatively, significant effort has been spent developing and designing portable gamma/beta devices [11,12] and new technologies for in-vivo detection of activity spatial and temporal distribution of radionuclide sources. These devices have been developed and tested to monitor the radioactive agent transit locally and determine the individual activity biodistribution. In our opinion, their adoption in MRT would permit an increase in the number of collected data points, thus reducing the uncertainties on dose-rate map estimation.

This study provides a critical overview of the currently available portable technologies, already tested for MRT or for brachytherapy (BT) treatment. These devices could be used in combination with nuclear medicine SPECT/PET imaging systems, representing the gold standard for in-vivo dose-rate map estimation and monitoring in MRT. Special attention is focused on systems that can help fulfill the emerging requirements of the recent European directive [13] and international recommendations for treatment personalization [14].

## 2. Materials and Methods

A Pubmed search was performed using the query string reported below to identify portable devices for in-vivo dosimetry measurements and monitoring radioactive agent transit and uptake in nuclear medicine treatments. Query: ((((“in-vivo dos*”) OR “internal dos*”) OR “time activity curve”) OR “uptake curve”) AND (((radionuclide therapy) OR molecular radiotherapy) AND detect*) NOT (external) AND “last 15 years”[PDat].

The research was restricted to the last fifteen years to include only the most recently published studies. The search was conducted on 15 January 2023. The bibliographies of retrieved papers and reviews were also examined to identify other relevant articles to be included. Only papers published in English were considered. Two authors independently reviewed titles and abstracts to decide on study inclusion. Full articles were considered eligible when reporting results of internal activity measurement or TIA determination in human subjects apart from SPECT or PET imaging systems. Papers describing dose-rate measurement from BT treatments were also been included. Indeed, BT is based on sealed radionuclide sources for which the effective decay is equal to the physical decay. Nonetheless, BT dose rate monitoring presents similar features to MRT (e.g., in dose gradient). Thus, the identified technology might be extended to MRT.

## 3. Results

Sixteen articles were retrieved reporting non-SPECT/PET based options for experimental dose/dose-rate monitoring in MRT or brachytherapy, including external probes, integration dosimeters and active detecting systems.

### 3.1. Identified Devices

The identified devices and their range of applications are summarized in Table 1 and described in terms of their features (typology, adopted technology, performance and application).

**Figure 1:**
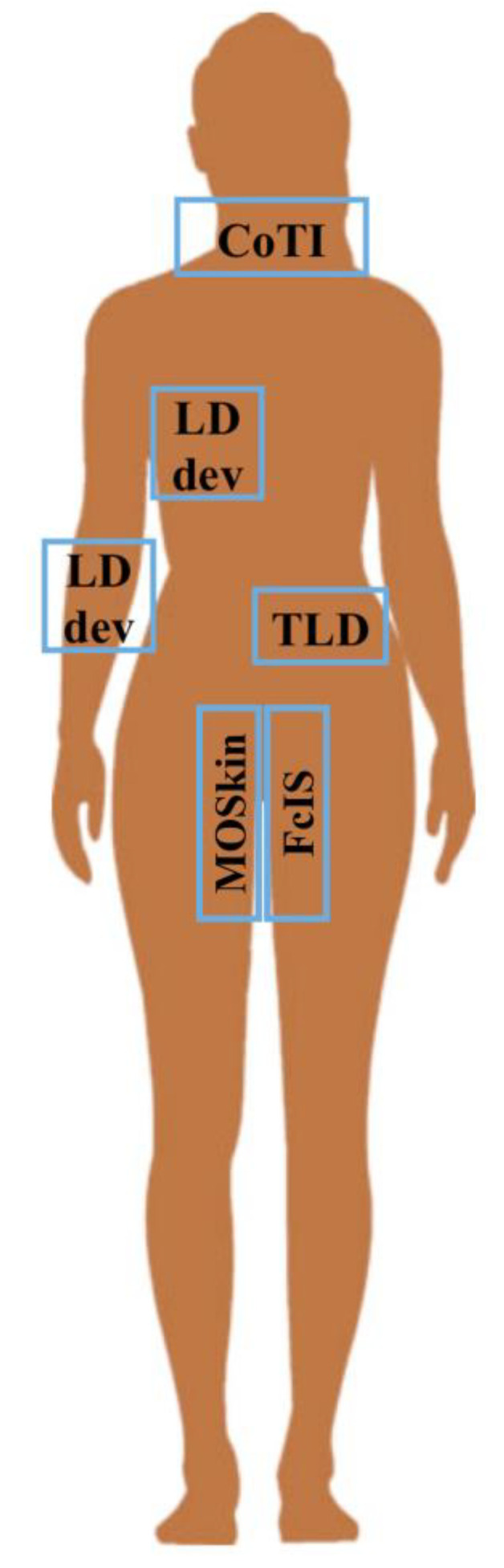
Sketch of the body sites investigated with dedicated devices or sensors described in the text.

Each device has been developed and tested to solve a clinical issue and, in some cases, as summarized in Figure 1, designed for specific body sites.

Their methodologies are presented, and performance, characteristics, limitations and uncertainties are discussed in the following.

#### 3.1.1. Thermo-Luminescence Dosimeters (TLD)

TLDs have been largely and successfully applied for integral absorbed dose evaluation. They are cheap, easy, portable, wearable and comfortable devices for simultaneously mapping several points on the body surface or accessible cavities. A TLD provides cumulative information on the exposure per point, so many TLDs should be placed in the same position and read out at fixed time intervals in order to reconstruct the exposure evolution over time. Results are elaborated offline and require a dedicated and relatively expensive reading-out system. Pre-irradiation and post-irradiation time-consuming cycles for annealing processes and great care in sensor handling are required.

In MRT, TLDs were adopted by Aschan et al. in 1999 [15] to test a method for the absorbed dose evaluation in critical organs. They were placed on the abdomen skin of patients treated with ^131^I and used for TIA determination in kidneys. The dosimeters were taped on each reference point for one hour, and a total of eight measurements were repeated, substituting the detectors in a time range of six days.

Most recently, TLDs have been used for in-vivo dosimetry in high-dose-rate (HDR, usually referred to dose delivered at rate >12 Gy/h and also much higher) brachytherapy. In the study presented by Gambarini et al. [16], TLDs were directly placed on a transrectal ultrasound probe and used for treatment evaluation of HDR-BT boosts of prostate cancer patients. The in-vivo integral absorbed dose measurements were compared with dose values calculated using the treatment planning system (TPS) along all the dosimeter positions. The robustness of the approach was confirmed at high-dose values for which the linearity of TLD was ensured.

#### 3.1.2. External Probes

External probes are commonly available in most nuclear medicine (NM) departments and are largely adopted to improve the appropriateness of local radio-protection rules to be applied during and after the end of the hospitalization. These systems allow the online registration of the average counts of the patients in anterior (AP) and/or posterior (PA) positions. They are usually portable but require dedicated operators (e.g., medical physicists, dosimetrists, or trained technicians) in order to perform specific measurements. In some departments, they are placed in the patient stay room in a fixed position (e.g., over the patient’s bed) to determine the average counts during the day. In these systems, the reproducibility is quite accurate, especially when relative calibration strategies are implemented.

The use of a portable external probe for ^131^I activity accumulation measurements in patients with differentiated thyroid cancer and hyperthyroidism was described by Bahamonde et al. [17]. The device consisted of a 7.6 × 7.6 cm^2^ NaI(Tl) scintillation detector, a cylindrical lead tube collimator with a steel cover and slots for attenuation lead plates at the end. The scintillation detector tube was mounted on a mobile mechanical support with adjustable height and was placed in the isolation room for measuring the radiation emitted by the whole body and thyroid of patients during their stay. Up to eight data points in four days were collected for TAC reconstruction and whole-body accumulated activity estimation. Saturation issues were experienced due to the high rate of radiation emitted from patients and the high detector sensitivity to gamma emissions.

#### 3.1.3. Portable Scintillators

Portable lightweight wearable monitoring systems were based on scintillation materials and photodetectors, which are promising options, since they provide extensive adaptability in shape and dimensions, battery powering possibility and cheapness.

Brinks et al. [18] and Abuqbeltah et al. [19] presented a Collar Therapy Indicator (CoTI) dedicated to measuring the uptake curve of patients undergoing ^131^I radioiodine therapy. Two gamma radiation detectors were placed in a disposable collar at predefined positions. The detector element consisted of a CsI(Tl) scintillation crystal and a silicon photo-multiplier (SiPM) with an active detection surface of 3 × 3 mm^2^. Data acquisition electronics within the detector analyzed the signal and produced the number of counts per second; then, the on-board processor communicated this information to the CoTI Control Unit for data storage. In the presented version, no energy discrimination was included.

The feasibility of the CoTI system was studied by measuring the individual thyroid uptake at 2 and 24 h after ^131^I administration (81 patients) and up to 96 h (8 patients) [19]. The uptake curve as a function of time was monitored for three clinically treated patients in a time range of 48 h from the administration [18]. Sequences of short measurements were preferable for continuous monitoring due to the discomfort experienced by patients wearing the collar. Repositioning the collar was possible with relatively good precision, also using a marker, although it might represent a source of uncertainties.

The accuracy of uptake measurements in patients who underwent imaging with ^123^I had been previously determined by comparing CoTI results with the planar gamma camera measurements. The detector’s non-linearity and lack of energy discrimination might result in an inaccurate estimation of the source activity. Dedicated correction factors and selecting appropriate energy ranges might reduce these inaccuracies.

Similar technology was adopted by Lucerno Dynamics (Cary, NC, USA), which developed a scintillating gamma sensor (in the following LDdev) based on a bismuth germanate crystal and photodetector [31] to assess the quality of ^18^F-FDG injections.

In the first clinical study of Williams et al. [20], three LDdevs were applied to subjects with locally advanced breast cancer and positioned over the subject’s palpable tumors, on their arms, and over their liver. The goal of the study was to provide dynamic measurements in the form of TAC during the tumor uptake period and compare them to the tumor-standard uptake value obtained by FDG PET images.

A second study was proposed by Lattanze et al. [21], who investigated the LDdev use for ^18^F-FDG injection quality assurance (QA) to check the abnormal FDG accumulation near the injection site and accurately determine accumulation and transit of the radioactive agent. Four LDdevs were adapted in adhesive pads topically applied on the skin (first proximal to the venous access site, second and third sensors on the contralateral arm and wrist, and the fourth over the liver) and read out by an on-board reader for data logging. The measurements for TIA determination were validated against dynamic PET images.

In both the studies mentioned above, TIA was determined in a limited time frame of 45/60 min from activity injection. LDdev resulted in a useful individual QA tool, although long-time TAC measurements might be affected by the patient’s motion changing the relative distance between the radioactivity and the sensor and between the arm with the sensor and other emitting areas of the body.

#### 3.1.4. MOSFET

The use of a metal-oxide-semiconductor field effect transistor (MOSFET) detector for in-vivo dosimetry has been extensively studied and well-established in external beam therapy. Zhen-Yu Qi et al. [32] proposed the use of miniature MOSFET detectors for dose verification of HDR-BT treatment planning. Indeed, the small dimensions of these types of detectors make them optimal candidates for BT since dosimeters can be easily placed within intraluminal catheters or special applicators can be inserted into patients.

Firstly, Haughey et al. [22] presented a dosimetry system based on a commercially available linear array MOSFET (LAM) detector fixed inside a modified sealed flatus tube, to be inserted into the rectum for rectal dose determination. This device was tested for ^192^Ir HDR-BT treatments of prostate needle implant patients and gynaecological patients with Fletcher suite implants. Authors complained about the difficulty in quantifying many uncertainties associated with MOSFETs, including calibration drift, angular dependence, and the inability to know the exact detector position during the treatment, which could be an important limitation in the presence of an extremely high dose gradient.

Then, Carrara et al. published two studies [23,24] to test a MOSFET dosimeter, named MOSkin, with a design optimized to operate in steep dose gradients. Two or three MOSkins were incorporated into a trans-rectal probe and arranged in a line to simultaneously measure the dose to the rectal wall at several spatial points. A lead radiopaque marker over the device made the dosimeter recognizable in CT images and its real position determinable. Moreover, in this case, the dosimeter was tested with both gynaecological and prostate BT treatments, and the measured doses were compared with the planned values. Although feasible, real-time dose measurement for online use has not yet been implemented. The critical issue, partially corrected by the marker, was related to the necessity of monitoring the dosimeter position with respect to the source since measurements could be affected by the rectum mobility and by the motion of the probe within the patient.

Successively, Jamalludin et al. [25,26] also considered MOSkin for comparison of measured to the planned dose from treatment planning in cervical cancer patients treated with ^60^Co HDR-BT. In this case a MOSkin was attached at the level of the third diode of a PTW probe type 9112 and inserted into the rectum, in 18 treatment sessions. The authors confirmed that a shift of the applicator or organ location seemed to be the main cause of deviations between planned dose and actual dose, affecting the dose uncertainty.

#### 3.1.5. Fiber-Coupled Inorganic Scintillators (FcIS)

Recent studies have highlighted five inorganic scintillators (ruby(Al2O3:Cr), Y2O3:Eu, YVO4:Eu, ZnSe:O, and CsI:Tl) for in-vivo measurements in HDR-BT [33]. Among the proposed inorganic scintillation detectors, Debnath and colleagues have shown that a scintillating detector made of 10 m silica (SiO2) optical fiber coupled with (Zn,Cd)S:Ag scintillating materials at the fiber apex exhibits the most favorable characteristics for time-resolved in-vivo dosimetry to be used in HDR-BT using two (water tank and solid water) phantoms [29].

Jørgensen et al. [27] used a detector based on a fiber-coupled inorganic scintillator for in-vivo dosimetry. The detector consisted of a detector probe (i.e., a 0.5 × 0.4 × 0.4 mm^3^ ZnSe:O crystal glued on to a 15 m-long optical fiber with a 0.5 mm core diameter) and an optical reader. The total probe diameter was 1 mm, allowing for the placement of the detector probe inside a standard brachytherapy catheter. The optical reader consisted of a photodiode unit, a data acquisition device, and a single-board computer (Raspberry Pi v3.0). The detector, previously characterized for dosimetry measurements in HDR and pulsed dose-rate (PDR)-BT [28,33], was placed in an additional brachytherapy catheter inserted in the prostate for determining the dosimetric impact of geometric variations in HDR prostate BT. Source tracking was performed on a catheter-by-catheter basis. The detector calibration was directly related to the accuracy of the radial catheter shift. A calibration uncertainty of less than 4.5% resulted in source-tracking errors smaller than 1 mm for source-to-detector probe distances of less than 45 mm. The source-tracking method detected only positional translations of the catheters in two directions and migration of the whole catheter implant could not be detected. Kaveckyte et al. reported a Monte Carlo characterization of high atomic number inorganic scintillators among prototype candidates for in-vivo dosimetry in HDR-BT [34], highlighting that high-Z crystals are sensitive to characterization and in-vivo measurement conditions. Moreover, the absorbed-dose energy dependence of high-Z scintillators was a function of the radial distance and the polar angle.

#### 3.1.6. WIDMApp

Finally, the attention of research and development in the matter of wearable sensors for IRD is focused on innovative combined systems such as WIDMApp (Wearable Individual Dose Monitoring Apparatus). Morganti et al. proposed in [30] a dosimeter system which combines a multichannel radiation detector worn by the patient during the entire MRT treatment for the in-vivo measurement of the patient-specific biokinetics; a simulation algorithm for modelling the proper anatomical structures and simulation of the particle interaction in the body, and a data processing tool for analyzing the registered signals, determines the TIAs at the organ level. The device is still under development.

Based on a Monte Carlo simulation, the study described by Morganti et al. presented a proof of concept for WIDMApp in the case of a 2.5 GBq ^131^I-radioiodine treatment and discussed the performance and the potentialities of the new approach. An anthropomorphic phantom with six emitting organ volumes (thyroid, two kidneys, bladder, liver and spleen) was simulated. The response of seven WIDMApp detector elements placed on the body surface was computed for a measurement period lasting 240 h from the activity administration. Then, using the WIDMApp data processing algorithm, the TIAs of the six source volumes were determined and compared with the simulated data. Accuracy at the level of 5% in the organ-based TIA determination was shown.

Despite the relative simplicity of the phantom’s anatomy and the radiopharmaceutical kinetics (only the uptakes of the mentioned organs were included in the simulation, and no contamination of the blood compartment was considered), the approach appeared fairly robust and flexible.

### 3.2. Cost and Portability

Several of the identified devices are commercially available (e.g., TLD, probe) while others are assembled using commercially available dosimeters (e.g., MOSFETs) or are prototypes (e.g., CoTI, FcIS). Thus, the cost of the devices described above is not so easily comparable. In addition, TLDs are reusable and cost less than 5–10 euros each, while MOSFET are reusable up to about 200 Gy and cost about 200 euros each. However, the cost of the readout system is about 10 times higher for TLDs than for MOSFETs. Nevertheless, the cost per patient depends on the number of performed in-vivo dosimetry, the type of identified dosimeter/read-out system and the duration of measurements related to the absorbed dose by the dosimeter. Regarding research detectors, the costs are not easily quantifiable since they depend on the technology readiness level (i.e., prototype, already engineered device, close/ready to market).

Regarding the portability, we selected only portable device with a weight of less than 10 kg and dimensions smaller than 30 × 30 cm^2^ (i.e., uptake portable probe), or wearable sensors (CoTI) or sensors included in catheters placed within patient body/natural cavities. However, the information reported in the manuscripts in terms of sensor geometry and technical specifications are limited and often difficult to quantify. It makes the devices not easily comparable under all circumstances.

Harmonization of reported information for each wearable/portable/insertable device would help selection of the optimal candidate for the specific application of MRT, and identification of the requirement gap(s) for each specific clinical use.

## 4. Discussion

Time-activity curves or dose rate maps determined on in-vivo measurements using nuclear imaging, blood counters, integrated dosimeters, or external probes are of great interest in MRT to assess individual dosimetry and to implement treatment personalization [35].

**Figure 2:**
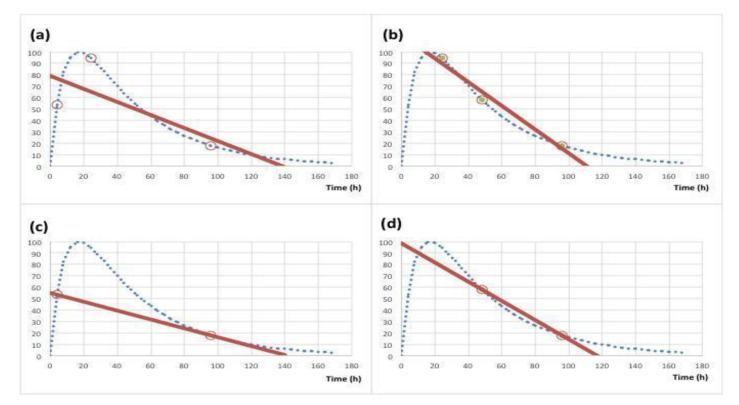
Example of the impact of data point sampling (4, 24 and 96 h vs. 24, 48 and 96 h, panel (**a**) and (**b**), respectively) or density (3 vs. 2 data points, panel (**a**) and (**c**) or panel (**b**) and (**d**), respectively) on the TAC estimation using a linear model (continuous line). The blue dotted line represents the expected theoretical TAC.

Data fitting is a common strategy for simplifying the mathematical description of an investigated biological system, and it could also simplify methods for online dosimetry and personalized MRT [36]. Based on available data, the fitting procedure could predict a radiopharmaceutical biokinetics behavior which is significantly different from theoretical ones. In particular, the image acquisition timing and the method used to interpolate data can influence the reconstruction of pharmacokinetic and tumor/organ clearance trends. If data points are scarce—due to the number of scans limited by reduced human resources and device availability—and do not properly span the radionuclide clearance time, the extrapolated TIA might vary within a wide range and, therefore, affect the absorbed dose calculation. A schematic image of the impact of data point sampling (4, 24 and 96 h vs. 24, 48 and 96 h) or density (2 vs. 3 data points) on the precision and accuracy in the curve reconstruction is reported in Figure 2.

In addition, the intrinsic uncertainty of instruments adopted for investigating the time-dependent biodistribution may introduce large uncertainties in the TIA estimation [37,38].

The voxel-based multimodel fitting method has been proposed and tested to be feasible on nuclear imaging patient data, leading to organ-based TIA estimations similar to the conventional ROI-based method [10]. These approaches take into consideration the spatial heterogeneity and image noise in the attempt to control fitting optimization and to provide robust results. However, the equality of pre-therapeutic and post-therapy biokinetics might not be applicable due to clinical patient conditions and cannot be observed due to the non-linear response of devices used in a wide range of activities.

Regarding diagnostic imaging, e.g., Renogram, quantitative analysis of such parameters as mean transit time and glomerular filtration rate requires an estimate of the plasma TIA, which is usually obtained with an external detection under the assumption that the plasma curve is proportional to the externally detected one [39]. Unfortunately, the estimation of the plasma TIA from an externally detected curve may be confounded by the detection of activity in the extravascular space. Thus, plasma TIA estimation using an external detection approach is liable to produce significantly biased results.

The availability of novel dedicated devices might significantly improve TIA accuracy in all the above scenarios.

Based on our search, several portable devices have been proposed and tested in clinical applications with various pros and cons for MRT or BT applications. The advantages and limits of the described devices are summarized in Table 2.

External probes and TLDs have been successfully applied in RNT for monitoring the exposure at a given point on the patient body surface. External probes are also used for monitoring patients in AP and PA at a relevant distance (e.g., 1 m) for radioprotection purposes [40]. Unfortunately, saturation effects are sometimes experienced, especially in the case of high-activity treatment, as in MRT. Moreover, these devices are often cumbersome and provide low portability, limiting their application outside of the NM departments.

On the other hand, TLDs can be fixed in defined positions on the body surface or adopted for intracavitary use but, passively operating, provide cumulative exposure measurements. In some cases, they are replaced during the treatment by a new annealing-treated TLD in order to provide sequential TIA measurements. However, the number of time points is limited and does not sufficiently span the TAC to accurately identify the uptake peak and reconstruct the entire long-term clearance behavior. In addition, time points require being elaborated offline using a dedicated and relatively expensive reading-out system.

Many of the remaining devices are based on scintillator technology, which allows for decreasing the detector dimension (up to millimetric size), also in the presence of high-activity sources. This feature permits the engineering of these detectors, opening the way to compact and wearable devices (e.g., CoTI [18,19]). This represents a unique opportunity to enable patients to do measurements her/himself with any medical or paramedical staff presence/intervention and to make long-time measurements feasible, thus improving the accuracy of TAC data point sampling. Electronics read-out still allows actively collecting TAC data points for up to a few days with a reduced time lapse between measurements. However, some devices are not yet optimized in terms of patient compliance. This aspect limits the possibility of wearing these devices all day long and during sleep time with the loss of information. For example, in the case of the CoTI tests where short measurement sessions were preferred with respect to continuous monitoring due to the feeling of discomfort experienced by patients wearing the collar. Furthermore, sources of the inaccuracy of the proposed setup, such as variations in thyroid size and position between patients, as well as the presence of hot and cold nodules within the thyroid, might affect the accuracy of dose calculations. The detector non-linearity might represent another source of the inaccuracy of the CoTI, along with the lack of energy discrimination of the detectors, resulting in the detection of scattered photons in addition to the measured primary photons.

The LDdev sensor [31] is an alternative option adopting a scintillator detector. It has been optimized for short-term TIA investigation, monitoring the local radionuclide transit and accumulation. In this case, the support of expert staff is required. The device arrangement and cabling limits patient comfort, and wireless systems are recommended for improving the device’s wearability and adding the possibility of analyzing data in real time. The availability of linearity of measurements over a longer time is still to be investigated and reported. However, the lack of shielding coverture around the device is currently the major limitation to enlarging its application in the MRT clinical practice since the response might be affected by further uptake of organ/tissue near the investigated emission point.

Aiming to significantly reduce the sensitive volume (up to order of 10^−6^ mm^3^), MOSFET (e.g., MOSkin [23,24,25,26]) or FcIS were adopted to integrate the detectors in intracavitary probes or catheters for BT dosimetric assurance. While this methodology results in great accuracy in dosimetric verification in steep dose gradients as in HDR/PDR-BT, the small dimensions make the sensitivity strongly affected by the change in the detector position with respect to patient anatomy, especially in high dose gradients. This issue was partially dealt with by equipping the dosimeter with a marker that could be identified on CT images. Multiple image-based strategies to monitor the actual probe localization in moving organs have to be implemented. Indeed, for TIA determination in MRT application, the very small dimensions of the detector could be a limitation in the presence of strong local inhomogeneity, for which a point-like sampling may not be representative of the averaged dosage in a given part of the investigated organ. This could be crucial in the attempt to improve data interpretation. Alternatively, several point measurements are needed to overcome this feature, although the number of channels needs to be balanced against the performance, the device portability, and the arrangement and cost of the electronics read-out system.

All the above-mentioned devices retrieved for this study investigate the TIA of a single organ or target tissue; otherwise, they provide cumulative information related to the whole body. This limitation is related to the measured signal that is accumulated in the same device or sensor. Contrary to this, the WIDMApp detecting system is composed of many wearable sensors that monitor the activity transit in different areas of the body surface contemporarily. Besides this relevant feature, the main innovation of the WIDMApp approach is the integration of a unique device for the detecting system and dedicated algorithms for patient-specific anatomical structure simulation and for the reconstruction of each radiation source (i.e., tumor and healthy radiopharmaceutical-uptaking organs) and particle interaction in the body and the sensors. Combining registered data with the simulated information, the WIDMApp analysis tool will be able to deconvolve the cumulative detected signal in contributions yielded by radiation emitted from different volumes according to the radiopharmaceutical biodistribution. It would be crucial for accurately determining the TIA at the organ/target level. The results of the feasibility study presented in [30] seem very promising, although the validity of the approach necessitates experimental tests on patients to confirm the assumptions.

Indeed, improving the accuracy and precision of the TIA determination and, consequently, the radionuclide treatment planning and dose estimation would enable the personalization of MRT and the individualization of the activity administration, with a high impact on patient outcome. For all these reasons, we believe that innovative dosimetric systems, contemporary monitoring all the emitting organs/tissues involved in the treatment, and being able to determine their proper TIAs accurately, merit investigation and a significant effort dedicated to their research and development.

This need is also targeted by the variety of funding opportunities in the field of radioprotection and treatment personalization (e.g., IAEA, EURATOM programs, PIANOFORTE partnership, NIH grants) potentially allowing different ideas to be tested, implemented, verified, and compared, addressing multiple technology readiness levels.

A possible limitation of this study is related to the wide variety of technologies actually applied to determine uptake, TIA and dose rate monitoring.

Another is the small number of patients involved in the identified studies. Moreover, several studies involve brachytherapy, which is not a form of MRT (no change in biodistribution with time) but nevertheless allows the appreciation of the dose rate change over time according to the physical half-time. In principle, this feature may demonstrate the proof of concept of its application to MRT.

## 5. Conclusions

A significant effort has been exerted over time to propose novel approaches and alternatives to SPECT/PET imaging acquisition and related methodology for TIA determination. Several portable monitoring systems have been designed and tested aimed at recovering the individual activity distribution from various radionuclides in both provisional and peri-treatment scenarios. The performance of these devices and potential improvements based on actual limitations have been discussed in this work. One of the most crucial issues is the lack of commercially available dosimetric systems that include dedicated algorithms to deconvolve the cumulative detector response and identify each emission point that contributes to the cumulative signal. This feature would allow the TIA determination of tumor and healthy organs separately with significant improvement of the dose estimation accuracy.

The development of new wearable and portable devices for in-vivo dosimetry and the design and development of specific simulation algorithms and data analysis tools to determine TIA at the voxel/organ/target level represents an important task to improve personalized treatment as well as to fulfil the novel European directive and international guideline requirements.

## Figures and Tables

**Table 1:** Portable devices for in-vivo dosimetry in treatments with radionuclides.

Device	Description and Adopted Technology	Operation Modality and Read Out System	Tested in MRT	Tested in HDR-BT	Investigated Body Site	N. Involved Patients	Monitored Time Range	Ref.
TLD	Cheap, easy, portable, wearable and comfortable devices. Thermo-luminescence detectors. BT: 3.2 × 3.2 × 0.9 mm^3^	Passive. Offline elaboration by dedicated external read out system.	(a) ^131^I thyroid treatment: TIA determination	(b) Prostate cancer HDR-BT: point dose measurement	(a) Kidney (b) Prostate	(a) 6 pts. (b) 4 pts	(a) 8 data points in 6 days	(a) [15] (b) [16]
External probe	Mobile gamma spectrometry system based on a 7.6 × 7.6 cm^2^ NaI(Tl) scintillation crystal	Active. Online registration of the average counts	TIA determination in whole body		Thyroid or Whole body	5 pts	Up to 8 data points in 4 days	[17]
CoTI	Wearable, lightweight collar with up to 4 gamma detectors: 3 × 3 mm^2^ CsI(Tl) scintillation crystal + SiPM	Active. Electronics read out on board transmits to external control unit for data logging	^131^I thyroid uptake measurement		Thyroid	(a) 3 pts (b) 89 pts	(a) Data points for 48 h (b) Data points at 2 and 24 h (81 pts) and up to 96 h (8 pts)	(a) [18] (b) [19]
LDdev	Scintillation crystal and photodetector	Active. Three/four sensors read out by a single box	(a) Routine 18F-FDG PET/CT imaging: TIA determination (b) Locally advanced breast carcinoma: tumour uptake		(a) Injection site, arms and liver (b) Tumour	(a) 21 pts. (b) 8 pts.	45/60 min	(a) [20] (b) [21]
LAM	Commercial linear array MOSFET detectors inserted in a flatus tube	Active. Integrated read-out electronics		Point dose determination for QA in (a) gynaecological or (b) prostate HDR-BT	Rectum	(a) 4 gynaecological pts (7 sessions in total) (b) 22 prostate pts (2 sessions)	Treatment time range	[22]
microMOSFET/ MOSkin	Trans-rectal probe with 2/4 customized MOSFET Dosimeter (sensitive volume: 4.8 × 10^−6^ mm^3^)	Active. Battery operated read-out system or directly connected to laptop		Point dose determination for QA in (a/c) gynaecological or (b) prostate HDR-BT	(a/b) Rectal wall (c) Rectum	(a) 9 pts (26 sessions) (b) 12 pts (18 sessions)	Treatment time range	(a) [23] (b) [24] (c) [25,26]
FcIS	The detector probe consisted of inorganic scintillator crystal glued onto a 15 m-long optical fibre with a 0.5-mm core diameter. The detector probe was placed in the prostate inside a standard BT catheter	Active. The optical reader consisted of a photodiode unit, a data acquisition device, and a single-board computer		Point dose for determining the dosimetric impact of geometric variations during HDR-BT	Prostate	(a) 18 pts (b) phantom	Treatment time range	(a) [27,28] (b) [29]
WIDMApp	Wearable multichannel detector and data processing system	Under development	^131^I thyroid: simulation study—not yet tested		Thyroid, Kidneys, Liver, Spleen, Bladder	NO	Treatment time range	[30]

Abbreviations: TLD: thermo-luminescence dosimeter; CoTI: Collar Therapy Indicator; LAM: linear array MOSFET; LDdev: Lucerno Dynamics device; MOSFET: metal-oxide-semiconductor field effect transistor; SiPM: silicon photo-multiplier; pts: patients; MRT: molecular radiotherapy; HDR: high dose rate; BT: brachytherapy; QA: quality assurance; TIA: time-integrated activity.

**Table 2:** Limits and advantages of portable dosimetry system for application in MRT.

Device	Advantages	Limitation Factors	Ref.
TLD	Low dependence on photon energy, dose rate and angular incidence of radiation. Allow several point mapping.	Only integral measurement. Uncertainty due to TLD substitution for TIA measurement. Relative expensive read out system and time-consuming process.	[15,16]
External probe	Patient can do measurement her/himself.	Cumbersome. No wearability and low portability. Require relative calibration strategy.	[17]
CoTI	Wearable and portable system. No presence of staff is required.	Long data taking pause at night due to feeling of discomfort experienced by patients wearing collar.	[18,19]
LDdev	Data are recorded on a on-board reader and exported afterwards.	Low comfort. Staff assistance is required.	[20,21]
LAM	Very small size. Dose rate independence.	Require frequent detector recalibration. Need sensor position. Angular and source energy spectrum dependent.	[22]
microMOSFET/ MOSkin	Optimized for high steep dose gradient. Use of markers to identify the actual dosimeter position in CT images. A correction of the angular dependence is necessary.	Need frequent single element calibration. Require very accurate detector element localization and orientation within patient.	[23,24,25,26]
FcIS	Optimized for high steep dose gradient.	The detector calibration is directly related to the accuracy of the radial catheter shift. A calibration uncertainty of less than 4.5% results in source-tracking errors smaller than 1 mm for source-to-detector probe distances of less than 45 mm. Moreover, some scintillators used in this research work have shown unstable scintillating intensity, significant afterglow, and require significant energy, temperature, and fibre signal attenuation corrections.	[27,28,29]
WIDMApp	Wearable and portable system. Simultaneous monitoring of all the emitting volumes involved. Very accurate TIA assessment (uptake and long-term retain). No constant presence of staff is required.	Under development/no-commercially available. Not yet tested on patients. No voxel-based.	[30]

## Data Availability

Not applicable.
